# A young child with pseudohypoaldosteronism type II by a mutation of *Cullin 3*

**DOI:** 10.1186/1471-2369-14-166

**Published:** 2013-07-31

**Authors:** Shoji Tsuji, Miyoko Yamashita, Gen Unishi, Reiko Takewa, Takahisa Kimata, Kiyoshi Isobe, Motoko Chiga, Shinichi Uchida, Kazunari Kaneko

**Affiliations:** 1 Department of Pediatrics, Kansai Medical University, 2-5-1 Shin- machi, Hirakata- shi, Osaka 573 1010, Japan; 2 Department of Pediatrics, Saiseikai Noe Hospital, Osaka, Japan; 3 Department of Pediatrics, Unishi Clinic, Osaka, Japan; 4 Department of Nephrology, Graduate School of Medical and Dental Sciences, Tokyo Medical and Dental University, Tokyo, Japan

**Keywords:** Pseudohypoaldosteronism, *Kelch-like 3*, *Cullin 3*, Gordon syndrome, Ubiquitination

## Abstract

**Background:**

Pseudohypoaldosteronism type II (PHA II), also referred to as Gordon syndrome, is a rare renal tubular disease that is inherited in an autosomal manner. Though mutations in *WNK1* and *WNK4* partially account for this disorder, in 2012, 2 research groups showed that *KLHL3* and *CUL3* were the causative genes for PHA II. Here, we firstly report on the Japanese child of PHA II caused by a mutation of *CUL 3*.

**Case presentation:**

The patient was a 3-year-old Japanese girl having healthy unrelated parents. She was initially observed to have hyperkalemia, hyperchloremia, metabolic acidosis, and hypertension. A close investigation led to the diagnosis of PHA II, upon which abnormal findings of laboratory examinations and hypertension were immediately normalized by administering thiazides. Genetic analysis of *WNK1* and *WNK4* revealed no mutations. However, analysis of the *CUL3* gene of the patient showed abnormal splicing caused by the modification of exon 9. The patient is currently 17 years old and does not exhibit hypertension or any abnormal findings on laboratory examination.

**Conclusions:**

In this patient, *CUL3* was found to play a fundamental role in the regulation of blood pressure, potassium levels, and acid–base balance.

## Background

Pseudohypoaldosteronism type II (PHA II), also referred as Gordon syndrome, is a rare renal tubular disease that is inherited in an autosomal manner [1]. Because thiazides are effective in the treatment of PHA II, a genetic defect in the NaCl cotransporter (NCC), the target transporter of thiazides, was thought to be the cause of PHA II. However, in 2001, Wilson et al. first reported that PHA II is caused by molecular abnormalities in 2 types of *WNK* (WNK; With No K [lysine]) genes, i.e., *WNK1* and *WNK4*[2]. Yang et al. confirmed that in *WNK4* knock-in mice, the NCC present in the cell membranes of the luminal surface cells of the distal tubule is excessively phosphorylated owing to a *WNK4* mutation, resulting in hypertension due to abnormal electrolyte/acid–base equilibrium and increased circulating blood volume [3]. However, a considerable number of patients with PHA II did not exhibit any genetic defect in *WNKs*. Recently, genetic defects in *Kelch-like 3 (KLHL3)* or *Cullin 3 (CUL 3)* were also reported to cause PHA II [4].

Here, we present the first case, to our knowledge, of PHA II caused by a mutation of *CUL3*, diagnosed in a 3-year-old Japanese child.

## Case presentation

### Direct sequencing of DNA and mRNA and splicing assay

Genomic DNA and RNA were extracted from peripheral blood lymphocytes by using a QIAamp DNA Blood Midi Kit (Qiagen, Venlo, The Netherlands) and Tempus™ Spin RNA isolation Kit (Applied Biosystems, Foster City, CA, USA), respectively. The mRNA was reverse-transcribed with oligo(dT) primer (Omniscript RT Kit, Qiagen, Venlo, The Netherlands). A 633-bp segment of the *CUL3* gene was amplified by polymerase chain reaction (PCR) (Primers: forward, 5′-TACGGAATAGAATTCCACTC-3′; reverse, 5′-CTCCATGAATGTATCCTGAC-3′) from genomic DNA. A 561-bp segment (390 bp, if exon 9 was skipped) was amplified by PCR (Primers: forward, 5′-TGAGGGAGCAAGGTAAAGCTC-3′; reverse, 5′-GCACCCGGACTGTAAGATCA-3′) from the cDNA. The PCR products were verified by sequencing. The ethics committee of Saiseikai Noe Hospital approved this study after consent from the patient’s guardian was obtained.

### Case report

**Figure 1:**
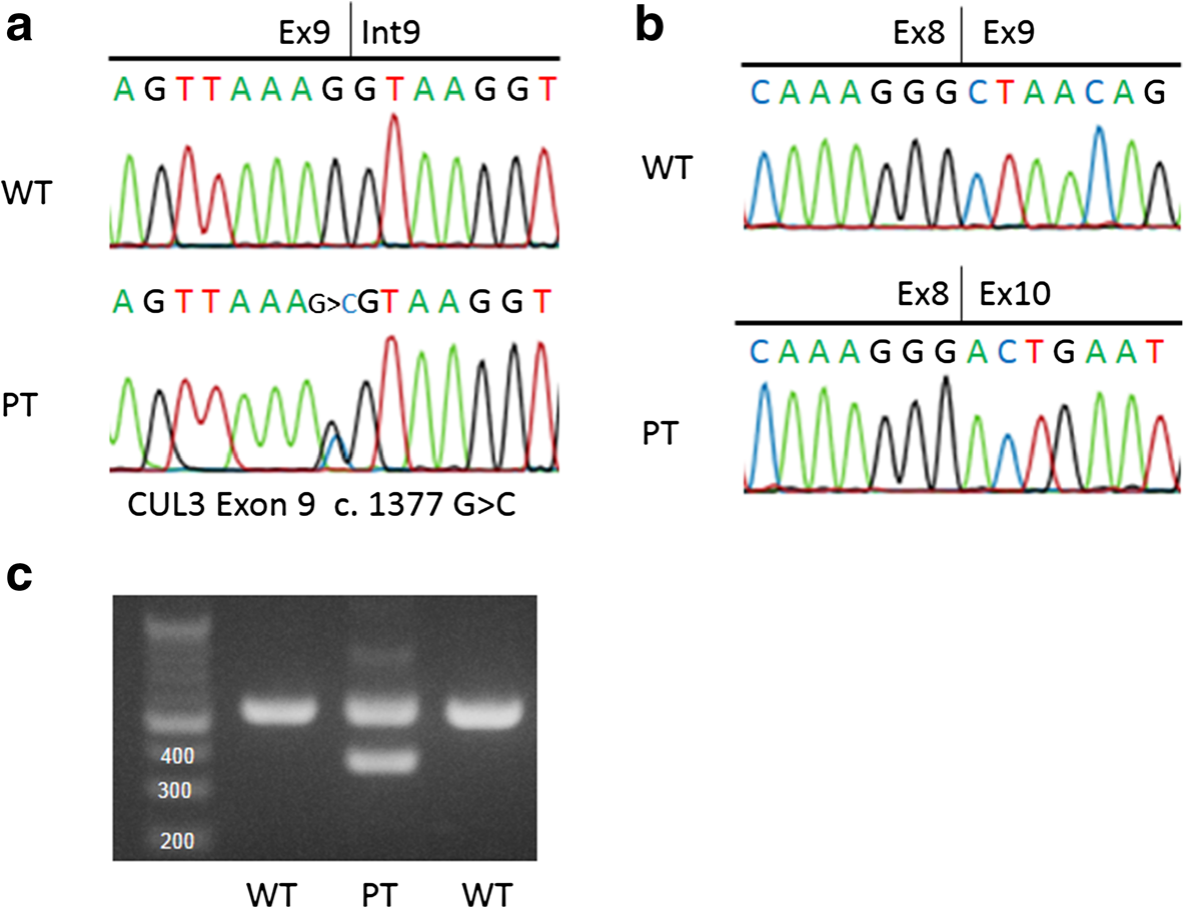
**Result of direct sequencing of DNA and mRNA and splicing assay. (a)** Sequence analysis of *Cullin 3* DNA in the index patient. In the patient (PT), the last guanine (G) of exon 9 of the *Cullin 3* in the wild type (WT) was displaced to cytosine (C). **(b)** Sequence analysis of *Cullin 3* mRNA in the index patient. In the wild type (WT), exon 8 is followed by exon 9 in Cullin 3 mRNA. In contrast, exon 10 is present just after exon 8 as exon 9 is skipped in the patient (PT). **(c)** Reverse Transcription Polymerase Chain Reaction (RT-PCR) of *Cullin 3* mRNA in the index patient. RT-PCR of *Cullin 3* mRNA demonstrated a smaller molecular weight band (390 bp) only in the patient (PT) in addition to the band (561bp) observed in the wild type (WT). The smaller band is considered a product of the skipping of exon 9.

The patient was a 3-year-old Japanese girl with healthy unrelated parents. Her perinatal history and past medical history were unremarkable. She was observed to have hyperkalemia, hyperchloremia, metabolic acidosis, and hypertension, when she was admitted to our hospital for the treatment of croup syndrome. An initial physical examination revealed normal growth, with a height of 95.5 cm (+0.3 standard deviation [SD]) and a body weight of 14.0 kg (+0.2 SD). The patient’s development was also normal. Her body temperature was 36.4°C. Chest auscultation revealed normal breathing sounds and a regular heart beat without murmurs. Examination of the patient’s blood and blood pressure revealed hyperkalemia (6.8 mEq/L), hyperchloremia (112 mEq/L), metabolic acidosis (pH, 7.248; HCO_3_^-^, 12.6 mEq/L), and hypertension (124/30 mmHg). The plasma renin activity (0.2 ng∙ml^-1^∙h^-1^) and plasma aldosterone concentration (25 pg/ml) were also low. A closer investigation led to the diagnosis of PHA II, and abnormal findings on laboratory examinations and hypertension were promptly normalized by the administration of thiazides. Subsequent genetic analysis of *WNK1* and *WNK4* revealed no mutations. The *CUL3* gene of the patient, her parents, and her younger brother were analyzed in 2013, based on the report by Boyden et al. [4]. Abnormal splicing caused by modification of exon 9 was detected only in the patient (Figure 1a). The skipping of exon 9 at the mRNA level was further confirmed by sequence analysis (Figure 1b) and reverse-transcription PCR (Figure 1c). Because a mutation of *CUL3* and manifestation of PHA II were not observed in the family members of the patient, in this case, the mutation was considered to be *de novo*. The patient is currently 17 years old and is healthy, with no hypertension or abnormal findings on laboratory examinations. The most recently recorded height and body weight of the patient were 155.6 cm (−0.4 SD) and 52.6 kg (−0.1 SD), respectively.

## Conclusions

Monogenic disorders such as PHA II give the opportunity to observe the clinical effects of a mutated gene. PHA II attracts special attention because its symptoms (hypertension, hyperkalemia, and acidosis) are usually caused by renal insufficiency, which is a relatively common cause of elevated blood pressure. PHA II is caused by the loss of WNK4-mediated tonic inhibition of thiazide-sensitive NCC in the distal convoluted tubule, by a kinase-dependent mechanism, due to loss-of-function mutations in *WNK4* and gain-of-function mutations in *WNK1*[2]. Though mutations in *WNK1* and *WNK4* are the cause of PHAII in some patients, recent successive studies have shown that *KLHL3* and *CUL3* are also causative genes for PHA II [4,5]. The CUL3 protein forms a complex with KLHL3 proteins, which then functions as a ubiquitin ligase. However, the exact role of these genes in PHAII is currently unknown. Recently, some groups reported that an interaction of KLHL3 with CUL3 and WNK4 induced WNK4 ubiquitination and reduced the WNK4 protein level, while a reduction in the interaction between KLHL3 and WNK4 attenuated the ubiquitination of WNK4, resulting in an increased level of the WNK4 protein [6-8].

The genotype-phenotype correlation in PHA II remains unknown, because only a few descriptions of PHA II in childhood have been reported [9-11]. Interestingly, Boyden et al. [4] reported that the onset of PHA II caused by *CUL3* mutation is earlier and the disease is more severe than is PHA II caused by *KLHL3* recessive, *KLHL3* dominant, *WNK4*, and *WNK1* mutations. In agreement with their report, the patient in the present case was diagnosed with evident hyperkalemic metabolic acidosis and hypertension at the early age of 3 years. It is noteworthy that despite the severity of the PHA II symptoms, administration of thiazides alone was sufficient to ensure normal development and growth of the patient.

This case demonstrates that *KLHL3/CUL3* plays a fundamental role in the regulation of blood pressure, potassium levels, and acid–base balance.

## Consent

Written informed consent was obtained from the patient for publication of this case report. A copy of the written consent is available for review by the Editor of this journal.

## Abbreviations

PHA II: Pseudohypoaldosteronism type II; NCC: NaCl cotransporter; KLHL3: *Kelch-like 3*; CUL 3: *Cullin 3*; PCR: Polymerase chain reaction.

## Competing interests

The authors declare that they have no competing interests.

## Authors’ contributions

ST and TK performed the literature search and drafted the manuscript. MY, GU and RT were also treating physicians for the patient. KI and MC carried out the molecular genetic studies and participated in the sequence alignmen. SU and KK conceived of the study and helped to draft the manuscript. All authors read and approved the final manuscript.

## Pre-publication history

The pre-publication history for this paper can be accessed here:

http://www.biomedcentral.com/1471-2369/14/166/prepub
